# Right Atrial Deformation Analysis in Cardiac Amyloidosis - Results from the Three-Dimensional Speckle-Tracking Echocardiographic MAGYAR-Path Study

**DOI:** 10.5935/abc.20180150

**Published:** 2018-09

**Authors:** Attila Nemes, Dóra Földeák, Péter Domsik, Anita Kalapos, Árpád Kormányos, Zita Borbényi, Tamás Forster

**Affiliations:** 2nd Department of Medicine and Cardiology Center, Medical Faculty, Albert Szent-Györgyi Clinical Center, University of Szeged, Szeged - Hungary

**Keywords:** Amyloidosis, Echocardiography, Three Dimensional / methods, Humans, Ventricular Dysfunction, Right, Speckle-Tracking

## Abstract

**Background:**

Light-chain (AL) cardiac amyloidosis (CA) is characterized by fibril deposits, which are composed of monoclonal immunoglobulin light chains. The right ventricle is mostly involved in AL-CA and impairment of its function is a predictor of worse prognosis.

**Objectives:**

To characterize the volumetric and functional properties of the right atrium (RA) in AL-CA by three-dimensional speckle-tracking echocardiography (3DSTE).

**Methods:**

A total of 16 patients (mean age: 64.5 ± 10.1 years, 11 males) with AL-CA were examined. Their results were compared to that of 15 age- and gender-matched healthy controls (mean age: 58.9 ± 6.9 years, 8 males). All cases have undergone complete two-dimensional Doppler and 3DSTE. A two-tailed p value of less than 0.05 was considered statistically significant.

**Results:**

Significant differences could be demonstrated in RA volumes respecting cardiac cycle. Total (19.2 ± 9.3% vs. 27.9 ± 10.7%, p = 0.02) and active atrial emptying fractions (12.1 ± 8.1 vs. 18.6 ± 9.8%, p = 0.05) were significantly decreased in AL-CA patients. Peak global (16.7 ± 10.3% vs. 31.2 ± 19.4%, p = 0.01) and mean segmental (24.3 ± 11.1% vs. 38.6 ± 17.6%, p =0.01) RA area strains, together with some circumferential, longitudinal and segmental area strain parameters, proved to be reduced in patients with AL-CA. Global longitudinal (4.0 ± 5.2% vs. 8.2 ± 5.5%, p = 0.02) and area (7.8 ± 8.1% vs. 15.9 ± 10.3%, p = 0.03) strains at atrial contraction and some circumferential and area strain parameters at atrial contraction were reduced in AL-CA patients.

**Conclusion:**

Significantly increased RA volumes and deteriorated RA functions could be demonstrated in AL-CA.

## Introduction

Systemic amyloidosis is a rare disease caused by the extracellular deposition of protein (amyloid) fibrils, which are composed of low molecular weight subunits (5 to 25 kD) of various serum proteins.^1^ The amyloid fibrils progressively damage the structure and function of the affected tissue, with variable clinical symptoms.^2,3^ For diagnosis of amyloidosis, biopsy from the affected tissue or from (abdominal) subcutaneous adipose tissue is necessary in most of cases.^4^ The classification of amyloidosis depends on the type of the precursor protein, including acquired monoclonal immunoglobulin light-chain amyloidosis (AL), wild-type or hereditary transthyretin amyloidosis (TTR), acquired serum amyloid type (AA) and other rare types. The mortality is especially high in light-chain (AL) amyloidosis.^5,6^

There are some warning signs that can draw attention to amyloidosis, such as nephrotic syndrome, tissue infiltration such as macroglossia, respiratory disease, carpal tunnel syndrome, bleeding, cachexia, haematological disease such as multiple myeloma, and genetic predisposition. As for the clinical signs, syncope is a poor prognostic factor and occurs quite frequently in patients with cardiac involvement.^7^ Cardiac involvement in amyloidosis varies according to the type of the disease.^1^ The real incidence of cardiac amyloidosis (CA) is not known precisely and is often diagnosed only during autopsy.^8^ Heart failure usually occurs in CA due to the combination of decreased myocardial compliance and compressed myocardial cells. These changes develop due to the infiltration by amyloid deposits and could lead to restrictive cardiomyopathy.^1^ Arrhythmias, pleural and pericardial effusion can also be detected in some cases.^4,9,10^ Although the right ventricle (RV) is mostly involved in CA, limited data is available about the involvement of the right atrium (RA).^11,12^ Therefore, this study aimed to characterize the volumetric and functional properties of the RA in AL-CA by three-dimensional (3D) speckle-tracking echocardiography (3DSTE).

## Methods

### Patient population

A total of 16 patients (mean age: 64.5 ± 10.1 years, 11 males) with biopsy-proven AL-CA were examined. Their results were compared to that of 15 age- and gender-matched healthy controls (mean age: 58.9 ± 6.9 years, 8 males). Baseline demographic characteristics of patients and controls are presented in Table 1. CA was defined in accordance with the current consensus criteria and practices.^6,13^ None of the patients with AL-CA was on anticoagulant treatment, but 2 of them received acetylsalicylic acid. Five patients received β-blockers, 7 patients were on angiotensine-converting enzyme inhibitors, while 11 patients took diuretics. The source of the biopsy was the bone marrow in 3 patients, the subcutis in 3 patients, the kidney in 5 patients, the heart in 3 patients, the gastrointestinal tract in 4 patients and the salivary gland in 1 patient. In 3 cases, samples were collected from more than 1 organ. In 11 out of 16 AL-CA patients, the diagnosis of multiple myeloma was confirmed. In 1 case, no treatment information was available. In all other cases, different types of chemotherapy or immunomodulatory treatment were administered. None of the healthy subjects in the control group had cardiovascular risk factors or any known diseases or received any medications. For cardiac evaluation, complete two-dimensional (2D) Doppler, tissue Doppler echocardiography, 3DSTE and N-terminal pro-B natriuretic peptide (NT-proBNP) level assessment were performed in all patients and controls. The present study was designed as a part of the **M**otion **A**nalysis of the heart and **G**reat vessels b**Y** three-dimension**A**l speckle-t**R**acking echocardiography in **Path**ological cases (**MAGYAR-Path**) **Study**. It has been organized to examine alterations in 3DSTE-derived parameters in different disorders compared to matched healthy controls among others (*magyar* means "Hungarian" in Hungarian language). The study protocol conformed to the ethical guidelines of the 1975 Declaration of Helsinki (and updated versions) and was approved in advance by the local institutional ethical committee. Informed consent was obtained from each subject.

**Table 1:** Baseline demographic and two-dimensional echocardiographic data in patients with cardiac amyloidosis and matched controls

	AL-CA patients (n = 16)	Controls (n = 15)	p-value
** Risk factors**			
Age (years)	64.5 ± 10.1	58.9 ± 6.9	0.08^*^
Male gender (%)	11 (69)	8 (53)	0.47^**^
Hypertension (%)	11 (69)	0 (0)	< 0.0001^**^
Diabetes mellitus (%)	1 (6)	0 (0)	0.46^**^
Hypercholesterolaemia (%)	6 (38)	0 (0)	0.02^**^
** Two-dimensional echocardiography**			
LA diameter (mm)	45.1 ± 6.7	36.6 ± 4.00	< 0.0001^*^
LV end-diastolic diameter (mm)	47.1 ± 5.7	47.3 ± 3.2	0.93^*^
LV end-diastolic volume (ml)	112.9 ± 31.9	104.9 ± 16.7	0.42^*^
LV end-systolic diameter (mm)	30.0 ± 5.3	30.4 ± 2.8	0.80^*^
LV end-systolic volume (ml)	41.7 ± 15.4	35.7 ± 6.9	0.11^*^
Interventricular septum (mm)	14.2 ± 1.9	10.4 ± 1.7	< 0.0001^*^
LV posterior wall (mm)	13.6 ± 1.7	10.4 ± 1.9	0.0003^*^
LV ejection fraction (%)	61.5 ± 11.9	65.7 ± 4.8	0.21^*^
E/A	1.71 ± 1.08	1.00 ± 0.45	0.14^*^

CA: cardiac amyloidosis; LA: left atrial; LV: left ventricular. Data expressed as mean ± standard deviation or or absolute numbers (percentage).

* Unpaired Student t test,

** χ^2^ test.

### Two-dimensional Doppler echocardiography

2D grayscale harmonic images were performed in the lateral decubitus position with using a commercially available ultrasound system (Artida^TM^, Toshiba Medical Systems, Tokyo, Japan) equipped with a broadband 1-5 MHz PST-30SBP phased-array transducer. During 2D Doppler echocardiography, chamber dimensions, volumes and ejection fraction were measured in accordance with the recommendations.^14,15^ Degree of mitral and tricuspid regurgitations was visually quantified by colour Doppler echocardiography.

### Three-dimensional speckle-tracking echocardiography

**Figure 1:**
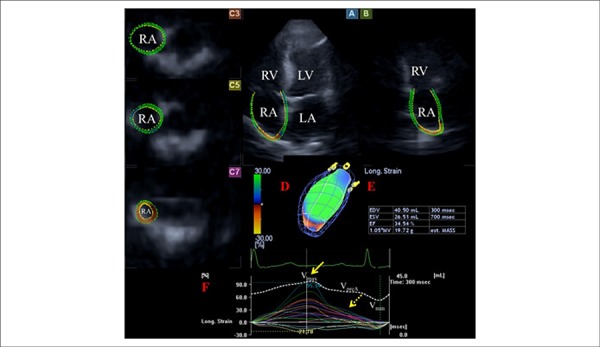
Images from three-dimensional (3D) full-volume dataset showing the right atrium (RA) are presented: apical four-chamber (A) and two-chamber views (B) and short-axis views at basal (C3), mid- (C5) and superior (C7) RA levels together with a virtual 3D model of the RA (red D) and with RA volumetric data (red E). Time - segmental (longitudinal) strain curves of all 16 RA segments (coloured lines) and a time - global RA volume change curve respecting cardiac cycle (white dashed line) are also presented (red F). Yellow arrow represents peak RA strain, while yellow dashed arrow represents RA strain at atrial contraction. V_max_, V_min_ and V_preA_ represent maximum and minimum RA volumes and RA volume at atrial contraction, respectively. LV: left ventricle; LA: left atrium; RV: right ventricle; RA: right atrium.

3D echocardiographic datasets were acquired with the same Toshiba Artida ultrasound system with a 1-4 MHz PST-25SX matrix phased-array transducer.^16^ After gain setting optimalisation, wide-angled pictures were recorded, in which 6 wedge-shaped subvolumes were acquired over 6 consecutive cardiac cycles during a single breath-hold. We used raw data format for further analysis. 3D Wall Motion Tracking software version 2.7 (Toshiba Medical Systems, Tokyo, Japan) was used for RA quantifications. Each 3D dataset was displayed in a 5-plane view: an apical 4-chamber (AP4CH) view, an apical 2-chamber (AP2CH) view and 3 short-axis views at different RA levels from the base to the apex. The examiner then set markers in the AP4CH and AP2CH views; in each plane, one marker was placed on the apex (superior region) and two other markers were placed at the edges of the tricuspid valve ring. As the next step, the software automatically detected the endocardium, and 3D wall motion-tracking analysis was performed through the entire cardiac cycle. During evaluations, RA appendage and the caval veins were excluded from the RA cavity (Figure 1).

From the acquired 3D echocardiographic datasets, time-global RA volume curves were created, allowing the measurement of maximum (V_max_) and minimum (V_min_) RA volumes and RA volume before atrial contraction (V_preA_). V_max_ was measured just before tricuspid valve opening at end-systole, while V_min_ and V_preA_ were measured just before tricuspid valve closure at end-diastole and at the time of P wave on ECG in early diastole, respectively. The systolic reservoir and diastolic passive (conduit) and active emptying (booster pump) phases of RA function were measured from the RA volumetric datasets:^17^

**Right atrial stroke volumes**
- Total Atrial Stroke Volume (TASV): V_max_−V_min_ (reservoir function)
- Passive Atrial Stroke Volume (PASV): V_max_-V_preA_ (conduit function)
- Active Atrial Stroke Volume (AASV): V_preA_−V_min_ (booster pump/active contraction function)

**Right atrial emptying fractions**
- Total Atrial Emptying Fraction: TASV/V_max_×100 (reservoir function)
- Passive Atrial Emptying Fraction: PASV/V_max_×100 (conduit function)
- Active Atrial Emptying Fraction: AASV/V_preA_×100 (booster pump/active contraction function)

Time-strain curves could also be created at the same time from the same 3D echocardiographic datasets. Unidirectional radial, longitudinal, circumferential and complex area and 3D strains could be also measured. Global strains were calculated by the software, which considered the whole RA, while mean segmental strains were obtained as the average of strains of 16 segments. A typical strain curve usually represents two peaks: the first peak indicates the reservoir phase, while the second peak shows characteristics of the booster pump phase of the RA function.^17^

### Statistical analysis

All continuous variables were presented as mean ± standard deviation. Categorical data were presented as frequencies and percentages (%). Comparisons among groups were performed by unpaired Student *t* test and χ^2^ test, when appropriate. Shapiro-Wilks test was used to test normal distribution in every dataset. Pearson correlation coefficient was calculated when needed. A 2-tailed *p* value < 0.05 was considered to indicate statistical significance. Reproducibility of 3DSTE-derived RA assessments has already been confirmed in a recent study.^17^ All statistical analyses were carried out using MedCalc software (MedCalc, Inc., Mariakerke, Belgium).

## Results

### Two-dimensional Doppler echocardiographic and NT-proBNP data

Significantly increased left atrial diameter, interventricular septum (IVS) and left ventricular (LV) posterior wall could be demonstrated in AL-CA patients as compared to matched controls (Table 1). Significant differences could be detected between AL-CA patients and matched controls in tricuspid annular plane systolic excursion (16.7 ± 3.1 mm vs. 20.0 ± 1.8 mm, p = 0.05) and RV fractional area change (32.3 ± 5.3% vs. 39.2 ± 3.5%, p = 0.04). Significant (≥ grade 3) mitral regurgitation could not be detected in any of the patients or control subjects. Only 1 patient with AL-CA had grade 4 tricuspid regurgitation. NT-proBNP level proved to be 9983 ± 11101 U/l in AL-CA patients.

### Three-dimensional speckle tracking echocardiographic data

Significant differences could be demonstrated in all RA volumes respecting the cardiac cycle. Total and active atrial emptying fractions were significantly decreased in AL-CA patients, while RA stroke volumes did not differ between the groups examined (Table 2). Peak global and mean segmental area strains proved to be reduced in AL-CA patients as compared to that of matched controls. Midatrial segmental circumferential, longitudinal and area strains, together with some basal strains, proved to be reduced in patients with AL-CA (Tables 3-4). Global longitudinal and area strains at atrial contraction were impaired in AL-CA patients, together with midatrial segmental circumferential and area strains (Tables 5-6). These results could suggest impaired longitudinal and circumferential RA function in the reservoir and active contraction phases of the RA function. Alterations in segmental RA strains could suggest non-uniformity of RA dysfunction in these cases.

**Table 2:** Comparison of 3DSTE-derived volumetric and volume-based functional right atrial parameters in patients with cardiac amyloidosis and in matched controls

	AL-CA patients (n = 16)	Controls (n = 15)	p-value
**Calculated Volumes**			
Vmax (ml)	85.0 ± 40.2	43.0 ± 13.2	< 0.0001^*^
Vmin (ml)	69.8 ± 37.3	30.8 ± 9.2	< 0.0001^*^
VpreA (ml)	79.2 ± 41.0	38.2 ± 12.8	< 0.0001^*^
**Stroke Volumes**			
TASV (ml)	15.2 ± 9.2	12.2 ± 7.3	0.40^*^
PASV (ml)	5.8 ± 5.1	4.8 ± 3.1	0.98^*^
AASV (ml)	9.4 ± 8.6	7.4 ± 5.9	0.47^*^
**Emptying fractions**			
TAEF (%)	19.2 ± 9.3	27.9 ± 10.7	0.02^*^
PAEF (%)	7.9 ± 8.0	11.5 ± 6.8	0.07^*^
AAEF (%)	12.1 ± 8.1	18.6 ± 9.8	0.05^*^

3DSTE: three-dimensional speckle-tracking echocardiography; CA: cardiac amyloidosis; AAEF: active atrial emptying fraction; AASV: active atrial stroke volume; PAEF: passive atrial emptying fraction; PASV: passive atrial stroke volume; TAEF: total atrial emptying fraction; TASV: total atrial stroke volume; Vmax: maximum right atrial volume; Vmin: minimum right atrial volume; VpreA: right atrial volume before atrial contraction. Data expressed as mean ± standard deviation.

* Unpaired Student t test.

**Table 3:** Comparison of 3DSTE-derived peak global and segmental peak right atrial strain parameters in patients with cardiac amyloidosis and in matched controls

	AL-CA patients (n = 16)	Controls (n = 15)	p-value
**Peak global strain**			
RS (%)	-13.8 ± 8.8	-15.1 ± 7.2	0.52^*^
CS (%)	7.1 ± 5.7	10.7 ± 9.6	0.21^*^
LS (%)	14.4 ± 9.8	21.9 ± 9.3	0.18^*^
3DS (%)	-6.9 ± 6.2	-8.1 ± 4.8	0.55^*^
AS (%)	16.7 ± 10.3	31.2 ± 19.4	0.01^*^
**Peak mean segmental strain**			
RS (%)	-17.1 ± 8.8	-18.9 ± 6.6	0.54^*^
CS (%)	12.2 ± 6.3	16.0 ± 9.2	0.19^*^
LS (%)	16.1 ± 9.3	24.2 ± 9.6	0.10^*^
3DS (%)	-11.5 ± 6.1	-12.6 ± 4.7	0.60^*^
AS (%)	24.3 ± 11.1	38.6 ± 17.6	0.01^*^

3DSTE: three-dimensional speckle-tracking echocardiography; CA: cardiac amyloidosis; 3DS: three-dimensional strain; AS: area strain; CS: circumferential strain; LS: longitudinal strain; RS: radial strain. Data expressed as mean ± standard deviation.

* Unpaired Student t test.

**Table 4:** Comparison of 3DSTE-derived peak segmental right atrial strain parameters in patients with cardiac amyloidosis and in matched controls

	AL-CA patients (n = 16)	Controls (n = 15)	p-value
RS basal (%)	-16.3 ± 10.2	-16.8 ± 5.7	0.87^*^
RS mid (%)	-14.9 ± 7.7	-18.5 ± 7.9	0.21^*^
RS superior (%)	-21.7 ± 16.5	-22.5 ± 11.9	0.87^*^
CS basal (%)	10.2 ± 4.9	15.1 ± 7.2	0.03^*^
CS mid (%)	7.9 ± 5.7	13.1 ± 6.9	0.02^*^
CS superior (%)	21.8 ± 16.7	20.8 ± 21.9	0.53^*^
LS basal (%)	17.6 ± 8.6	24.4 ± 13.4	0.19^*^
LS mid (%)	18.0 ± 13.3	30.7 ± 13.1	0.01^*^
LS superior (%)	10.9 ± 10.5	16.8 ± 9.9	0.07^*^
3DS basal (%)	-11.6 ± 7.2	-11.2 ± 5.3	0.86^*^
3DS mid (%)	-9.6 ± 5.6	-12.0 ± 5.9	0.24^*^
3DS superior (%)	-14.3 ± 10.8	-15.4 ± 9.3	0.77^*^
AS basal (%)	19.9 ± 9.1	30.1 ± 12.7	0.02^*^
AS mid (%)	21.9 ± 15.9	41.0 ± 15.4	0.002^*^
AS superior (%)	34.4 ± 30.9	47.9 ± 48.3	0.66^*^

3DSTE: three-dimensional speckle-tracking echocardiography; CA: cardiac amyloidosis; 3DS: three-dimensional strain; AS: area strain; CS: circumferential strain; LS: longitudinal strain; RS: radial strain. Data expressed as mean ± standard deviation.

* Unpaired Student t test.

**Table 5:** Comparison of 3DSTE-derived global and segmental peak right atrial strain parameters at atrial contraction in patients with cardiac amyloidosis and in matched controls

	AL-CA patients (n = 16)	Controls (n = 15)	p-value
** Global strain at atrial contraction**			
RS (%)	-6.4 ± 6.7	-6.2 ± 6.1	0.93^*^
CS (%)	10.6 ±11.9	7.8 ± 8.5	0.47^*^
LS (%)	4.0 ± 5.2	8.2 ± 5.5	0.02^*^
3DS (%)	-2.8 ± 4.9	-3.6 ± 4.4	0.62^*^
AS (%)	7.8 ± 8.1	15.9 ± 10.3	0.03^*^
** Mean segmental strain at atrial contraction**			
RS (%)	-8.5 ± 6.0	-8.5 ± 4.8	0.99^*^
CS (%)	5.3 ± 6.2	8.6 ± 7.3	0.10^*^
LS (%)	6.5 ± 4.0	9.0 ± 5.7	0.20^*^
3DS (%)	-5.3 ± 4.6	-6.2 ± 4.5	0.57^*^
AS (%)	11.2 ± 6.8	17.2 ± 12.3	0.11^*^

3DSTE: three-dimensional speckle-tracking echocardiography; CA: cardiac amyloidosis; 3DS: three-dimensional strain; AS: area strain; CS: circumferential strain; LS: longitudinal strain; RS: radial strain. Data expressed as mean ± standard deviation.

* Unpaired Student t test.

**Table 6:** Comparison of 3DSTE-derived segmental right atrial strain parameters at atrial contraction in patients with cardiac amyloidosis and in matched controls

	AL-CA patients (n = 16)		Controls (n = 15)	p-value
RS basal (%)	-9.6 ± 9.4		-8.5 ± 5.7	0.72^*^
RS mid (%)	-7.2 ± 5.5		-7.5 ± 4.5	0.89^*^
RS superior (%)	-9.0 ± 8.2		-10.2 ± 7.5	0.67^*^
CS basal (%)	4.6 ± 4.4		10.1 ± 10.5	0.07^*^
CS mid (%)	3.6 ± 4.4		8.4 ± 6.2	0.02^*^
CS superior (%)	10.6 ± 11.9		7.4 ± 9.8	0.42^*^
LS basal (%)	7.7 ± 4.0		8.9 ± 6.3	0.53^*^
LS mid (%)	6.8 ± 6.9		10.6 ± 7.3	0.12^*^
LS superior (%)	4.1 ± 5.7		7.0 ± 7.7	0.20^*^
3DS basal(%)	-5.4 ± 7.0		-6.0 ± 5.2	0.81^*^
3DS mid(%)	-4.2 ± 4.1		-6.0 ± 4.5	0.27^*^
3DS superior (%)	-6.8 ± 6.9		-7.1 ± 6.5	0.90^*^
AS basal (%)	9.9 ± 5.4		16.0 ± 10.6	0.06^*^
AS mid (%)	9.4 ± 9.1		18.2 ± 12.4	0.03^*^
AS superior (%)	15.9 ± 19.8		17.2 ± 22.3	0.87^*^

3DSTE: three-dimensional speckle-tracking echocardiography; CA: cardiac amyloidosis; 3DS: three-dimensional strain; AS: area strain; CS: circumferential strain; LS: longitudinal strain; RS: radial strain. Data expressed as mean ± standard deviation.

* Unpaired Student t test.

## Discussion

Among several types of amyloidosis, in AL amyloidosis is characterized by fibril deposits, which are composed of monoclonal immunoglobulin light chains and is mainly associated with B-cell type diseases, like clonal plasma cell or other B-cell dyscrasias.^18^ The course of the disease can be progressive in case of cardiac involvement.^19^ The main cause of death in patients with AL amyloidosis is cardiac involvement leading to heart failure or arrhythmias, and is considered to be an important prognostic factor. Without cardiac presentation, the survival is 4 years,^20^ in some cases, it is only 8 months.^21^

In case of cardiac involvement, typically concentric ventricular thickening with RV involvement, biventricular function with normal or near normal ejection fraction and valvular thickening can be seen.^22,23^ The speckled or granular myocardial appearance is characteristic of amyloid deposit, but the absence of this phenomenon is not rare.^2^ Disproportionate septal deposition can mimic hypertrophic cardiomyopathy with dynamic LV outflow tract obstruction. Atrial thrombus is common, especially in AL-CA, and sometimes is associated to atrial fibrillation. Diastolic dysfunction is the earliest echocardiographic sign and can often be detected before any clinical symptom.^24,25^ The end-diastolic thickness of the IVS is > 12 mm in the absence of any other cause of LV hypertrophy in heart involvement.^13^ In CA, the thickness of the LV wall is not in correlation with the course and outcome of the disease.^6^ Doppler myocardial imaging measures of the RV can identify early impairment of cardiac function or stratify risk of death in patients with AL-CA.^26^ Impaired RV function was found to be a predictor of worse prognosis of early mortality in AL-CA.^27^ However, detailed analysis of AL-CA-associated RA volumetric and functional alterations was not documented.

With 2D echocardiography, the assessment of RA is limited due to viewing dependency and geometric difficulties. Regularly, RA diameter and area are measured in AP4CH view.^14,15^ 3D echocardiography is a new clinical modality that allows the accurate measurement of atrial phasic volume changes.^12,16,17^ Moreover, several functional properties, including stroke volumes and emptying fractions and strains at different phases of the cardiac cycle, could be measured at the same time from the same 3D dataset, allowing detailed analysis of the RA during 3DSTE.^16,17^ In the present study, over increased RA volumes in all phases, alterations in emptying fractions and strains characterizing systolic reservoir, and late-diastolic active booster pump RA functions could be demonstrated. These findings could be explained by infiltration of the atrial wall with amyloid fibrils, impaired left and/or right heart failure, effects of cardiovascular risk factors, haemodynamic reasons, local fibrosis or oedema. In a recent study, severe LA dysfunction could be demonstrated in AL-CA, therefore the role of LA-RA interactions could also not be excluded.^28^ Segmental RA strain analyses showed RA regional differences, suggesting their different contributions to RA (dys)function, as mentioned before (non-uniformity of RA dysfunction). Further studies are needed to confirm our findings in a larger population, comparing results to other diseases with LV hypertrophy as well. It should also be examined whether the demonstrated pattern of RA dysfunction is specific or not for AL-CA, and whether it has or not a diagnostic or prognostic value.

### Limitations

The limited number of patients with AL-CA is one of the most important limitations of the study. However, biopsy-proven amyloidosis with cardiac involvement is a rare disease. Although the atrial septum is part of both atria, it was considered to be part of the RA in this study.

## Conclusions

Significantly increased RA volumes and deterioration in RA functions could be demonstrated in AL-CA - theoretically due to infiltration of the atrial wall with amyloid fibrils - but other causes, including haemodynamic reasons, cannot be excluded.
